# Paths to the discovery of antivenom serotherapy in France

**DOI:** 10.1186/s40409-016-0074-7

**Published:** 2016-06-08

**Authors:** Rosany Bochner

**Affiliations:** Laboratory for Scientific and Technological Information on Health (LICTS), Institute for Communication and Scientific and Technological Information on Health (ICICT), Oswaldo Cruz Foundation (FIOCRUZ), Av. Brasil, 4365 – Prédio Haity Moussatché, 2° andar, sala 206, 21045-360 Rio de Janeiro, RJ Brazil; National Toxico-Pharmacological Information System (SINITOX), Av. Brasil, 4365 – Prédio Haity Moussatché, 2° andar, sala 206, 21045-360 Rio de Janeiro, RJ Brazil; Post-Graduate Program in Health Information and Communication (PPGICS), ICICT, Av. Brasil, 4365 – Prédio Haity Moussatché, 2° andar, sala 206, 21045-360 Rio de Janeiro, RJ Brazil

**Keywords:** Calmette, Phisalix, Antivenom serotherapy, Snake, History of science

## Abstract

The current study presents a descriptive chronological survey of the articles published by Césaire Auguste Phisalix and Albert Calmette on snake poison, with the aim of shedding a light on the areas of research and reasoning followed by these scientists, leading up to their simultaneous discovery of antivenom serotherapy in 1894. The path taken by Phisalix is revealed in 15 articles that demonstrate the motivation of a naturalist and the way he confronted the puzzle of immunity against snake venom. In the case of Calmette, two articles preceded the discovery; microbiology was his theoretical base and the Pasteurian spirit of solving health problems his driving force. These two researchers followed distinct paths, mobilized by different motivations, but produced one single result. It is incontestable that the discovery of antivenom serotherapy was the work of two groups of researchers who deserve equal recognition, but who, in fact, did not receive it. Following the discovery both Calmette and Phisalix returned to their previous motivations. Calmette put the discovery into practice and began to produce antivenom serum in Lille. He came to be generally considered as the sole discoverer of antivenom serotherapy and was the recipient of a number of prestigious prizes. Phisalix, on the other hand, received little recognition and returned to his original interests, devoting himself to research on natural immunity. In Brazil, the discovery of antivenom serum therapy had a profound impact on the work of Vital Brazil Mineiro da Campanha, a researcher known worldwide for his scientific discoveries and for the evidence of the specificity of antivenom serums.

## Background

The discovery of antivenom serotherapy, which occurred in France, was presented to the French Society of Biology on the 10th of February 1894 by representatives of two Parisian research institutions. The organizations were the National Museum of Natural History, represented by Césaire Auguste Phisalix and Gabriel Bertrand, under the direction of Jean Baptiste Auguste Chauveau, and the Pasteur Institute, represented by Albert Calmette, under the direction of Emile Roux [1, 2].

A number of scientific events, both in the area of microbial therapy and in the specific subject of animal venom associated with immunization and natural immunity, created a fertile ground for this discovery to occur. Concerning microbial therapy, Bon [3] mentions only the identification of diphtheria and tetanus toxins, and the discovery of serum therapy as major findings. However, the following events were also important for the development of this field:

- the first vaccination against smallpox by Edward Jenner on May 14, 1796 [4];
- Pasteur’s virus attenuation and vaccination against avian cholera in 1880 [5];
- vaccination against anthrax by Louis Pasteur, Charles Chamberland and Émile Roux in 1881 [6];
- discovery of the tuberculosis bacillus by Robert Koch in 1882 [7];
- vaccination against rabies by Louis Pasteur in 1885 [8];
- development of immunity against *Staphylococcus pyosepticus* after peritoneal transfusion observed by Jules Héricourt and Charles Richet in 1888 [9];
- identification of the diphtheria toxin by Émile Roux and Alexandre Yersin in 1888 [10];
- publication of immunity studies by Metchnikoff in 1889 [11];
- identification of the tetanus toxin by Knud Faber in 1890 [12];
- discovery of antitoxins by Emil Von Behring and Shibasaburo Kitasato in 1890 [12].

Regarding venoms, immunization and natural immunity, Bon [3] mentions only works related to immunization: those of the Italian Domenico Fornara [13], the English Henry Sewal [14] and the French Maurice Kaufmann [15]. The following events, however, are worthy of recognition in this context:

- verification of the natural immunity of vipers and non-venoms snakes against viper venom by Felice Fontana in 1781 [16, 17];
- identification, in *Vipera berus* venom, of a protein called “vipérine” or “échidnine” similar to digestive enzymes by Lucien Bonaparte in 1841–1843 [16, 18];
- the discovery of active alkaloid principles in the poisons of frogs and salamanders by Pierre Louis Gratiolet and Stanislas Cloëz in 1852 [16, 19];
- the immunization of dogs against frog venom by Domenico Fornara in 1877 [16, 20];
- the extraction of an alkaloid from the venom of *Salamandre terrestre* by Zalesky in 1886 [16, 21, 22];
- the development of resistance in pigeons against the venom of *Crotalus* snakes by means of the injection of increasing doses of the venom by Henry Sewall in 1887 [14];
- the development of the theory that successive inoculations of low doses of venom may provoke a certain degree of immunity against higher doses by Maurice Kaufmann in 1889 [23]; and
- proof that successive inoculations of small amounts of venom may produce resistance, but are incapable of conferring true immunity against envenomation, by Maurice Kaufmann in 1892 [15].

It is incontestable that the discovery of antivenom serotherapy was the work of two groups of researchers who deserve equal recognition, but who, in fact, did not receive it. Even though in 1894 the French Academy of Sciences bestowed the Montyon Prize on Césaire Auguste Phisalix and Gabriel Bertrand for their discovery [24], and in 1898 gave the Bréant Prize to Césaire Auguste Phisalix for the sum of his work on venom and poisonous animals [25], it was in reality Albert Calmette who received all the recognition for the discovery. Several scientific studies – such as those by Abelardo Sáenz [26], Arlindo de Assis [27], Bernard [28–30], Bernard and Nègre [31], Chung and Biggers [32], Gernez-Rieux [33], Guénel [34], Klobusitzky [35], Leake [36] and Rivière and Bon [37] –indicate Calmette as the unique discoverer of antivenom serotherapy, leaving Phisalix in the most profound anonymity. Many factors contributed to this situation: the premature death of Phisalix in 1906, at only 54 years of age; the departure of Gabriel Bertrand from the National Museum of Natural History in 1900 to work on other areas in the Pasteur Institute in Paris; and the efforts of Calmette, in contrast to those of Phisalix, in advertising his work at home and abroad. Moreover, and most importantly, Calmette began to produce antivenom serum at the Pasteur Institute in Lille, putting his discovery into practice. For his part, Phisalix returned to his original interests and dedicated himself to his studies on natural immunity.

Within the context of this simultaneous discovery, a question arises: how is it that two groups from distinct institutions made the same discovery at the same time?

In order to address this question, the present work undertakes a descriptive and chronological analysis of the articles published by Césaire Auguste Phisalix and Albert Calmette on the subject of animal venom, with the aim of presenting their motivations and the process of reasoning followed by these researchers, which led them to the simultaneous discovery of antivenom serotherapy.

### The work of Césaire Auguste Phisalix on animal venom before the discovery of antivenom serotherapy

Phisalix published his first work on animal venom in August 1889 in the journal *Association française pour l’avancement des sciences*. This first one and the four subsequent articles were published between 1889 and 1891. One of them was written in collaboration with P. Langlois and another with Charles Louis Contejean. These studies were related to the poison of the terrestrial salamander and mentioned:

- the efficacy of the poison lethal dose was dependent on the mean of inoculation into the organism;
- immunity could be developed though administration of successive doses of poison;
- the non-refractory state of the salamander with respect to its own poison;
- the effect of salamander poison on the nervous system, temperature, breathing and movement of mammals;
- the two types of cutaneous glands (mucous and specific); and
- the type of secretion, whether acid or alkaline [22, 23, 38–40].

Afterwards, Phisalix began to study frog venom and in 1893 published, with Gabriel Bertrand, his first two works on the subject. They concluded that frog blood contained active substances possessing the same physiological properties as its poison, but at a low concentration. These active elements, products of the internal secretion of the cutaneous glands, would be responsible for the relative immunity of the frog to its own poison [41, 42].

In August 1893, Phisalix published a work on the toxicity of the blood of the terrestrial salamander, in which he concluded that it contains a toxin similar to that secreted by the poison glands and which reaches the blood by means of an internal secretion mechanism [43].

In December of the same year, in partnership with Gabriel Bertrand, Phisalix began his studies on the toxicity of snake blood. The two explained their choice of this animal based on the existing opposition between the composition and properties of this venom and those of frog and salamander poison. They concluded that snake blood contains some principles analogous to those of the venom, which are endowed with great physiological activity and stem from internal secretions of the glands. The presence of these toxic principles in the blood must be considered the true cause of snake immunity against its own venom [44].

In January 1894, Phisalix and Bertrand, both influenced by the naturalist vision, returned to the subject of natural immunity. They published a work on the presence of venom glands in non-venomous snakes and the toxicity of their blood. They observed that there are toxic substances analogous to “échidnine” in the blood of non-venomous snakes, which is the result of the internal secretion of the upper labial glands, and that the physiological and chemical similarity of these principles to “échidnine” explains the immunity of these animals to viper venom [45].

Still in January 1894, Phisalix and Gley published an article about the effects of thyroid removal on salamanders, in which they concluded that this procedure could, in the case of inferior invertebrates, produce serious effects and even death [46].

In spite of Phisalix’s medical training, it is important to emphasize that in these early 11 studies, he approached only topics related to naturalists’ interests. His motivation was derived from animal biology to the detriment of human therapeutics.

Nevertheless, a new direction came to be followed in his research at the beginning of 1894. At this time, Phisalix and Bertrand returned to their studies on the toxicity of viper blood and its venom. In the article published in the *Archives de physiologie normale et pathologique*, they confirmed that viper venom is in part destroyed or weakened by heat in accordance with the intensity and duration of heat exposure. They also suggested an analogy between venom and microbial toxins when they stated: “the porcelain filters hold the toxic material just as they hold microbial toxins” 1 [47]. They concluded that there are in viper blood toxic principles analogous to those of venom, which have the same chemical and physiological properties. Consequently, viper immunity to its own venom is due to an internal secretion from specific glands, in such a manner that its active principles impregnate the organism and create a familiarity with large doses of this powerful venom [47].

On February 5, 1894, Phisalix and Bertrand presented their experiments on the weakening of viper venom by heating and the vaccination of a guinea pig against it. They confirmed that the heated venom becomes a vaccine while small doses of the venom in its natural state provoke only a progressive and slow familiarity, but not a genuine immunization. They also came to the following conclusions: there are two toxic substances in the venom, one acts in a manner comparable to certain diastases, and to which they gave the name “échidnase”. The other substance, which they called “échidnotoxine”, has a strong general effect on the nervous system, disrupting the functioning of the vasomotor apparatus to the point of causing death. When given to guinea pigs, it produces serious hypothermia. Both two substances can be considerably altered, if not destroyed, by temperatures of around 75 °C [48].

Following this, Phisalix and Bertrand deepened their research on the causes of natural immunity in non-venomous snakes. They assumed that the immunity of these animals to viper venom resulted from the presence in their blood of toxic principles analogous to those of the venom. Such substances are also found in the upper labial glands of non-venomous snakes and are not only homologous to the venom glands of the vipers, but are indeed analogous, at least regarding the internal secretion [49].

It is important to note that before February 5, 1894, when Phisalix announced his findings on the weakening of viper venom by heating and the vaccination of a guinea pig against this venom, he had never explicitly shown any interest in therapy or for the treatment of cases of envenomation.

It is possible that his interest was awakened following a conversation with Elie Metchnikoff, as Albert Calmette states in a letter to his parents from February 13, 1894: “Two weeks ago, just after my first communication about the toxicity of snake blood, Messrs. Phisalix and Bertrand, two scholars of the museum (said to be quite bad experimenters) asked Metchnikoff at the Biology Society if I could supply them with a small quantity of snake venom. Metchnikoff replied to them that my work was almost completed, that I probably had no venom left. In any case, it was of little use to devote time to work on this question because my research was very advanced, that I had discovered not only immunization of animals, but also the cure, etc. – Three days later, Phisalix and Bertrand read a note to the Academy of Sciences in which they reproduced for their own benefit what Metchnikoff had told them, and announced that they had found a way to immunize animals against poisoning with the help of heated venom” 2 [50].

On the other hand, it is important to point out that Phisalix was not only a naturalist. He was trained in medicine and by 1894 he had already published several works on microbiology, two of which deserve to be mentioned as they are related to the influence of heat on the sporogenesis of *Bacillus anthracis* [51, 52]. It is quite probable that the idea of using heat to attenuate snake venom had its origins in these two works.

### The work of Albert Calmette on venom before the discovery of antivenom serotherapy

Calmette began his study on snake venom in October, 1891. At that time, he was in Saigon, where he was responsible for opening a new branch of the Pasteur Institute. In a letter dated November 7, 1891, Calmette explained to his parents his motivation to take up the study of this topic: “Twelve days ago, I launched into the most interesting experimental research on the venom of the famous snakes called Najas or capel cobras that every year kill, in India alone, according to the official statistics of the English government, 21,000 people! In Cochinchina, these snakes are rarer. In any case, there is a good number: a village near Bac-Lieu was invaded by a multitude of snakes that were fleeing from floodwaters. The snakes entered homes and bit 40 people, of which four died almost immediately. An Annamite, part snake charmer and part wizard, was able to take control of 19 of these animals. The district administrator, an intelligent man, upon learning of this, telegraphed me to ask if I wanted him to send the snakes to my laboratory, along with the Annamite who knew how to handle them. Naturally, I accepted immediately, and the snakes arrived in a barrel. Fourteen of them were still alive. I kept three in cages and killed 11 in order to extract their venom glands; with these glands I made a quantity of preparations that allowed me to obtain venom that is pure, easy to conserve, and that I have used in very interesting experiments, because never before has it been possible to preform such a complete study of venom under such favorable conditions as I have been able to gather” 3 [53].

Calmette’s motivation seems to have been inspired by the Pasteurian spirit of the age, dedicated to the elimination of health problems, as well as carrying out research that could confer recognition and prestige in the international scientific community [54].

In March, 1892, Calmette published his first work on snake venom, in *Annales de l'Institut Pasteur*, in which he dealt with the following topics:

- the preparation and conservation of venom;
- the physiology of envenomation, covering natural immunity;
- the means of venom injection;
- the non-transmissibility of envenomation by the blood;
- the physical-chemical properties of venom;
- the action of antiseptics and other chemical substances;
- attempts to produce artificial immunity against evenomation, without any success.

Nevertheless, he concluded that it was possible to cure envenomated animals by neutralizing the venom absorbed by blood with the help of subcutaneous injections of gold chloride into the wound itself, around it and in the upper part of the affected limb. He declared that this treatment, applied to a human being, would produce the same results. He believed that the efficacy of this treatment would likely extend to bites of all venomous snakes, since the diverse “échidnines” (*vipérine*, *crotaline*, *najine, élaphine*, etc.) demonstrated slight differences in physiological action amongst themselves. Furthermore, all authors who had conducted experiments with the venom of exotic snakes were in agreement that the venom of cobra is the most active [55].

In 1892, Calmette received only a single honorable mention, in the Barbier Prize from the French Academy of Medicine, for his work on the curative power of gold chloride in treating envenomation by *Naja tripudians* or *cobra capel*. This study was discussed in his first article [55]. The reasons for his losing the prize were explained as follows: “The bite of this snake is terrible and is responsible for the deaths, every year, of too great a number of our soldiers in Cochinchina. Mr. Calmette would have won the prize if the efficacy of gold chloride had been demonstrated. Unfortunately, the results that he offered are not convincing” 4 [56].

It was only on January 13, 1894, that Calmette published his second work on venom, in which he analyzed the toxicity of *cobra capel* blood. In this article, he cited the research of Phisalix and Bertrand on the toxicity of viper blood [44]. He confirmed that *cobra capel* blood is extremely toxic, that the symptoms of envenomation are the same as those produced by inoculation with venom, that the blood of the cobra is not venomous for toads, fish, and not even for a small species of non-venomous snake. He also stated that the non-venomous snake appeared to be completely resistant to the venom, in the same way as the cobra, and that the injection of small non-lethal doses of pure cobra blood did not confer any immunity to animals, which succumbed to inoculations of venom given later [57].

### Phisalix’s crossword puzzle

The cumulative character of science has often been described as solving a giant crossword puzzle, in which each piece represents a unit of scientific knowledge, with new pieces being placed in the existing structure [58, 59].

The line of reasoning followed by Phisalix, which lead to the discovery of antivenom serotherapy, may be interpreted as solving a small crossword puzzle made of four pieces. The first of these was based on Phisalix’s two articles on the effect of heat on the sporogenic property of *Bacillus anthracis* [51, 52] and was related with the use of heat to remove toxic properties from toxic microorganisms. The second was based on his work on the comparative toxicity of the blood and the venom of the viper, in which the analogy between venom and microbial toxins was verified [47]. The sum of the information in both articles lead to the fact that viper venom is in part destroyed or weakened by heat in conformity with the intensity and duration of the heating. The third piece stemmed from the work on the weakening of venom by heat and the vaccination of guinea pigs against this venom [48]. It was suggested, in that research, that heated venom becomes a vaccine and its use in small doses in its natural state produces only a progressive familiarity, but not a true vaccination. The fourth and final piece was derived from the work dedicated to the causes of natural immunity to viper venom in non-venomous snakes [49]. In estimating that this immunity results from the presence in the blood of toxic principles analogous to those in the venom, Phisalix and Bertrand went beyond the results achieved by Sewall in 1887 and Kaufmann in 1889, who got as far as immunization of animals, but did not proceed with the topic of treatment with antivenom serotherapy [14, 23]. They described the transfer of immunity from one animal to another (passive immunity), paving the way for the discovery of antibodies.

### Calmette and microbiology

The information contained in the two works by Calmette on venom published before the discovery of antivenom serotherapy is not sufficient to provide an understanding of his line of reasoning [55, 57]. That being the case, it is necessary to look at his biography, as well as his other published works, in order to understand the path to his discovery.

Any understanding of Calmette’s training in microbiology should begin with the period between 1888 and 1890, which he spent in Saint Pierre and Miquelon. While there, in a self-taught way and based on a few published texts at that time, he performed experimental research on *rouge de morue* (the appearance of a reddish coloration in cods because of contamination by a microorganism). Calmette thought of this research as his self-initiation into microbiological methodology, in which he isolated the microorganism, identified the conditions under which it develops, the fact that it originates in salt, and even that a small quantity of sodium sulfite prevents its proliferation. He was unable, however, to demonstrate the toxicity of the microorganism, as the consequences of the phenomenon were purely commercial, given that no one wanted to buy the cod when it changed color [54].

Calmette arrived in Paris in 1890 to participate in one of the first microbiology courses offered by the Pasteur Institute, taught by Dr. Émile Roux. His three-month stay at the institute marked an important stage in his life: full of enthusiasm and admiration for the Pasteurian discoveries, he decided to abandon his career in naval medicine and dedicate himself to research. It did not take long for him to gain the confidence of Émile Roux, who introduced him to Louis Pasteur. The latter chose Calmette to found a laboratory in Saigon dedicated to the preparation of vaccines for smallpox and rabies. It was there that Calmette would begin his work on snake poison [59].

According to Calmette, at the time he began his studies on *Naja* venom, limited data on its physiology were available. Only some of its properties had been explained in the work of Weir Mitchell and Reichard in the USA, Wall and Armstrong in England, Armand Gautier and Maurice Kaufmann in France and, especially, in the admirable volume of Sir James Fayer (*Thanatophidia indica*), published in London in 1872 [18].

Although Calmette had not completely mastered the field, which may explain the future controversies that surrounded him, Phisalix and Vital Brazil [20], he believed that he could not miss an excellent opportunity to study a topic in which interests had increased following the discoveries by Émile Roux and Behring on the diphtheria and tetanus toxins [18]. For Calmette, venoms and envenomations represented a topic that physicians had rarely focused on, but interests in it had been awakened because of the close connections discovered between microbial toxins and problems of immunity. He followed a therapeutic approach and discovered the antivenoms in the wake of German, Japanese, English and French microbiologists [4–12].

Thus, Calmette’s solid experience in microbiology must have prevailed, providing him a glimpse of the analogy between microbial toxins and venom.

## Conclusions

The careful, chronological analysis of the work of these researchers, along with their biographical information, helps to understand the lines of reasoning adopted by each of them.

It is possible to recognize how these two distinct groups, coming from two important French institutions of the period, and arising from different motivations and paths, came to the same discovery at the same time.

In addition, we should not forget to mention the fertile ground that existed at the time of the discovery, thanks to the work of numerous researchers, as much in the area of microbial therapy as in the more specific field of immunization and natural immunity.

In conclusion, the analysis of the work of Albert Calmette and Césaire Auguste Phisalix still serves to confirm the effervescence of this field at the time, given that a great number of their precursors were found to be explicitly cited in their work.

In Brazil, the discovery of antivenom serum therapy had a profound impact on the work of Vital Brazil Mineiro da Campanha (1865–1950), a researcher known worldwide for his scientific discoveries and for his evidence of the specificity of antivenom serums. He was also responsible for the creation of the Butantan Institute in São Paulo city, São Paulo state, and the Vital Brazil Institute in Niteroi, Rio de Janeiro state [60].

Sharing the work of these researchers, especially that of Césaire Auguste Phisalix, has become mandatory in order to ensure that history is told in a more fair and trustworthy way.

## Footnotes

1 Original text: "les filtres de porcelaine retiennent la matière toxique du venin comme ils retiennent les toxines microbiennes".

2 Original text: "Il y a 15 jours, à la suite de ma 1ère communication sur la toxicité du sang des serpents, MM. Phisalix et Bertrand, deux savants du Muséum (réputés comme assez mauvais expérimentateurs) ont demandé à M. Metchnikoff à la Société de Biologie si je ne pourrais pas mettre à leur disposition un peu de venin de cobra. M. Metchnikoff leur répondit que mon travail était presque terminé, que je ne devais plus avoir beaucoup de venin, et que d’ailleurs il était inutile de se mettre aussi à travailler cette question des venins car mes recherches à moi étaient très avancées, que j’avais trouvé non seulement l’immunisation des animaux mais même la guérison etc. – Trois jours après, Phisalix et Bertrand faisaient à l’Académie des Sciences la lecture d’une note dans laquelle ils reproduisaient pour leur propre compte ce que leur avait dit M. Metchnikoff, et ils déclaraient avoir trouvé le moyen d’immuniser les animaux contre le venin à l’aide d’injections de venin chauffé".

3 Original text: "Depuis une douzaine de jours je suis lancé dans des recherches expérimentales on ne peut plus intéressantes sur le venin de ces fameux serpents qu'on appelle des Najas ou cobras capel et qui tuent chaque année, dans l'Inde seule, d'après les statistiques officielles du gouvernement anglais, 21.000 personnes! En Cochinchine, ces serpents passent pour plus rares. Or voici ce qui prouve que, dans tous les cas, il en existe un bon nombre : un village des environs de Bac-Lieu, presque envahi par les eaux s'est vu assaillir par une foule de cobras fuyant devant l'inondation. Les cobras entraient dans les maisons et ont piqué paraît- il 40 personnes dont 4 étaient mortes presque aussitôt. Un annamite moitié psylle et moitié sorcier s'est emparé de 19 de ces animaux. L'administrateur de l'arrondissement, homme intelligent, informé du fait m'a aussitôt télégraphié pour me demander si je voulais qu'il m'envoie au laboratoire les cobras et l'annamite qui a su les prendre. J'ai accepté aussitôt, bien entendu, et les serpents me sont arrivés dans un baril. 14 étaient encore vivants. J'en ai gardé 3 dans des cages, et j'en ai tué onze pour extraire leurs glandes à venin; avec ces glandes j'ai fait une quantité de préparations qui m'ont permis d'obtenir du venin pur, facile à conserver, et je l'emploie à des expériences dont l'intérêt est très grand parce que jamais une étude aussi complète du venin n'a pu être tentée dans des conditions aussi favorables que celles que j'ai pu réunir".

4 Original text: "La morsure de ce serpent est redoutable, et fait périr chaque année en Conchinchine un trop grand nombre de nos soldats. Aussi M. Calmette aurait-il obtenu un prix si l’efficacité du chlorure d’or eût été démontrée. Malheureusement les preuves qu’il fournit ne sont pas convaincantes".
